# An overview of artificial intelligence based automated diagnosis in paediatric dentistry

**DOI:** 10.3389/froh.2024.1482334

**Published:** 2024-12-24

**Authors:** Suba B. Rajinikanth, Densingh Samuel Raj Rajkumar, Akshay Rajinikanth, Ponsekar Abraham Anandhapandian, Bhuvaneswarri J.

**Affiliations:** ^1^ Faculty of Medicine, Srilalithambigai Medical College and Hospital, DR MGR Educational and Research Institute, Chennai, India; ^2^ Department of Traumotology & Orthopedics, Kursk State Medical University, Kursk, Russia; ^3^ School of Computer Science and Engineering, VIT University, Vellore, India; ^4^ Department of Prosthodontics, Thai Moogambigai Dental College and Hospital, Dr. M.G.R. Educational and Research Institute, Chennai, India; ^5^ Department of Periodontics, Sri Balaji Dental College and Hospital, BIHER, Chennai, India

**Keywords:** artificial intelligence, convoluted neural network, paediatric dentistry, deep learning, automated diagnosis, dental caries, dental plaques

## Abstract

Artificial intelligence (AI) is a subfield of computer science with the goal of creating intelligent machines (1) Machine learning is a branch of artificial intelligence. In machine learning a datasets are used for training diagnostic algorithms. This review comprehensively explains the applications of AI in the diagnosis in paediatric dentistry. The online database searches were performed between 25th May 2024 to 1st July 2024. Original research studies that focus on the automated diagnosis or predicted the outcome in Paediatric dentistry using AI were included in this review. AI is being used in varied domains of paediatric dentistry like diagnosis of supernumerary and submerged teeth, early diagnosis of dental caries, diagnosis of dental plaques, assessment of bone age, forensic dentistry and preventive oral dental healthcare kit. The field of AI, deep machine learning and CNN's is an upcoming and newer area, with new developments this will open up areas for more sophisticated algorithms in multiple layers to predict accurately, when compared to experienced Paediatric dentists.

## 1 Introduction

Artificial intelligence (AI) is a field of science and engineering concerned with the computational understanding of what is commonly called intelligent behaviour, and with the creation of artefacts that exhibit such behaviour (1). AI is a process by which a computer is made to help healthcare professionals in various aspects of recognition, prediction and suggesting a solution for a problem, by creating complex layered algorithms called the convoluted neural networks (CNN). Artificial intelligence (AI) is a subfield of computer sciences to create machines that match human intelligence (1).

Machine learning is a branch of artificial intelligence which aids to the development of automated, sophisticated, and objective algorithmic techniques for data analysis (2). Machine learning is a branch of artificial intelligence. In machine learning a datasets are used for training diagnostic algorithms (2). The algorithm when fed with a similar data, is programmed to generate an analysis for prediction or diagnosis, which already has stored similar datasets as memory (2). The dataset already available is used to compare and evaluate the newly developed model (2). Machine learning is used to complement the clinical decisions when large and complex data is involved. The quantity and quality of training datasets and neural networks decides the accuracy of prediction. Thus such good quality datasets can be used to produce AI algorithms which can help to predict and diagnose in dental issues in children.

Deep learning (DL), as a subfield of machine learning, allows computational models consisting of numerous layers, to learn representations of data characterized by varying degrees of abstraction (3). The large amount of high quality data is fed into a system about a particular domain which acts as the memory equivalent of a human brain. These systems are designed to support health-care workers in their daily duties and with tasks that rely on the manipulation of data and knowledge (4). So, when a question is raised with an input like imaging, the AI uses the deep machine learning process and picks up a similar image and gives an answer.

Considering the above, the primary objective of this review is to raise awareness about the novel applications of artificial intelligence in the field of paediatric dentistry. The recent publications on the field of AI and deep learning application in the field of paediatric dentistry along with the challenges and limitations are going to be summarized. Moreover, published data on novel robotically approaches in the management in paediatric dentistry are going to be analysed.

## 2 Methods

The Online Database search was conducted in PubMed, Embase and Scopus using the keywords “(Automated diagnosis) AND (Paediatric dentistry)”, “(“Artificial Intelligence” OR “Machine Learning”) AND “Paediatric dentistry” “and” (Robotics) AND (Paediatric Dentistry)”. These searches were performed between 25th May 2024 to 1st July 2024. To be included in the review, studies needed to focus on either the automated diagnosis of Paediatric dentistry using deep learning algorithms. Studies without English manuscript or clinical relevance were excluded.

### 2.1 Criteria for eligibility

Inclusion criteria

• Original Articles
• Required Keywords present
• Time period January 2017 to April 2024
• English language

Exclusion criteria

• Articles without the keywords
• Articles where full-text versions are unavailable
• Ongoing studies.

## 3 AI networks in automated diagnosis—an overview

**Figure 1:**
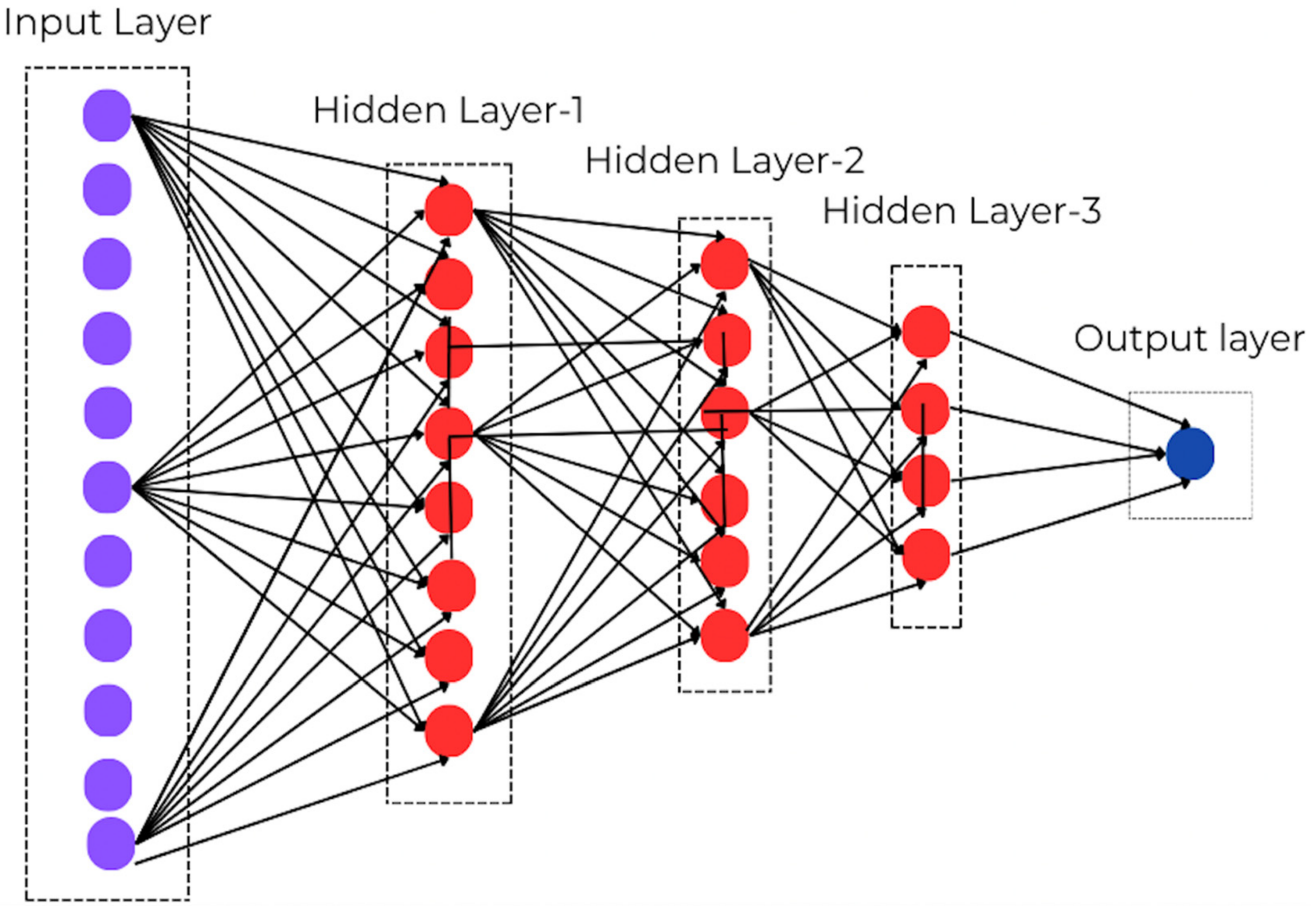
The neural network of machine learning.

The AI algorithms can analyse vast datasets of diagnostic data, medical images, and patient history to improve diagnostic accuracy and identify diseases earlier. Neural network is a subset of machine learning, and which is at the heart of deep learning algorithms. The neural network consists of nodes, which has an input layer, one or more processing layers in the middle and an output layer as illustrated in Figure 1. Every node has a certain threshold, and it gets activated if the output of any individual node is above the specified threshold value. Thus sending data to the next layer of the network, or else no data is passed on to the next level.

**Figure 2:**
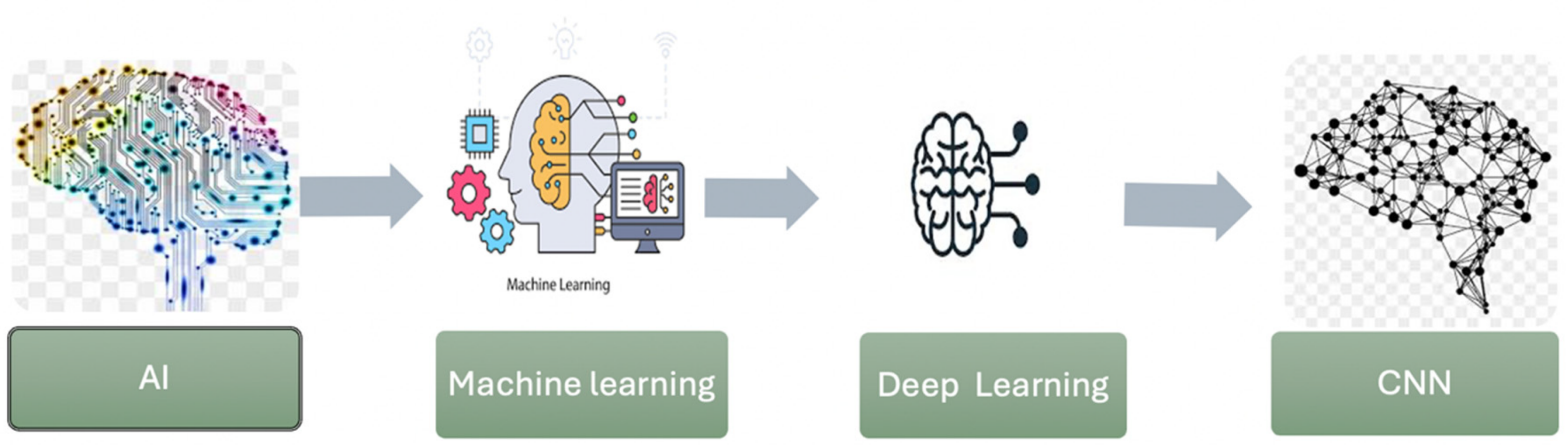
AI networks in automated diagnosis.

A subset of deep learning algorithm made up of several layers (Figure 2), known as Convoluted neural network recognises images using three-dimensional data for classification and object recognition. The design architecture of CNN is inspired by visual processing human brain. CNNs are widely used in areas such as image classification, object detection, facial recognition, and medical image analysis & interpretation. Using the Inclusion criteria 15 research papers were included in this mini review. A comparison of several studies was made based on the techniques used and results were compared, as in Table 1.

**Table 1:** Tabulation of results and AI techniques used in the selected 15 articles.

S. No.	Authors	Domain and technique studied	Results
1	Caliskan et al. (5)	AI-based CNN model—classification of submerged primary teeth on OPGs	Satisfactory results, Reproducible to that of experts.
2	Tuzoff et al. (6)	Faster R-CNN-based architectures—teeth detection and numbering	Automated diagnosis can save time and help in maintenance of electronic records.
3	Kılıc et al. (7)	Identification of deciduous teeth on OPGs and uses In digital forensic dentistry.	Time-saving tool
4	Kaya et al. (8)	YOLO V4—tooth detection and numbering	used as a pre-processing tool for detection of dental pathologies (8)
5	Sadegh-Zadeh et al. (10)	Ten different machine learning modelling techniques and two assessment methods (Leave-One-Out and K-fold) were used (10)	MLP, RF, and SVM (kernel = RBF) were the best performing machine learning models which could accurately detect the risk up to 97%.
6	Zheng et al. (2)	CNNs (three types) compared for the diagnosis of caries and pulpitis	Multimodal CNN was accurate and precise
7 &8	Ramos-Gomez et al. (12) and Karhade et al. (13)	Questionnaire based applied ML for detection of caries	Early identification of Childhood caries was possible.
9	Zaborowicz et al. (14)	Neural modelling for estimation of chronological age of children using radiographic images	Near accurate results
10	Bunyarit et al. (15)	Dental maturity ratings based on Demirjian's scores by artificial neural network (ANN) computational technique	Accurate results
11	Lee et al. (16)	ML algorithms using datasets of radiographs for age estimation	More efficient compared to traditional method (16).
12	Wang et al. (17)	Developed a tool kit which consisted of a short form (SF) to assist parents in evaluating their children's oral health status and need for treatment, which conceptualized health as having physical, mental, and social components.	The primary objective of the toolkit was to help the dentist with dental examinations (17).
13	Gajic et al. (18)	Comparison of Statistical methods and artificial intelligence algorithms in oral health on adolescent quality of life	Comparable results of human intuition and machine algorithms as both agreed on how the responses should be split.
14	You et al. (19)	AI model for diagnosis of dental plaque by the caretakers.	Clinically accurate prediction levels
15	Joesph et al. (20)	A Laser Induced Auto Fluorescence (LIAF) based probe with AI program to detect dental Plaques.	Early detection of plaques helps to maintain good oral health

### 3.1 AI in characterisation and diagnosis of submerged and supernumerary tooth

Caliskan et al. (5) in their pilot study, using deep learning and convolutional neural network (CNN) algorithms (Faster Region-based CNN architecture), processed a radiograph to define the boundaries of submerged molars. A separate testing set was used to evaluate the diagnostic performance of the system and compare it with that of experts in the field (5). The system was extremely accurate in its performance in comparison with observers (5).

Tuzoff et al. (6) developed a faster R-CNN-based architectures for automatic teeth detection and numbering. Although AI based algorithm saves time and aids in diagnosis, this study also demonstrated that the teeth detection errors produced by the system both false positives and false negatives as the limitations of AI in the diagnosis of teeth detection and numbering.

Kilic et al. (7) in their study developed an artificial intelligence (AI) algorithm (Cranio Catch, Eskisehir-Turkey) using Faster Region-based CNN Inception v2 (COCO) models to automatically detect and number deciduous teeth as seen on paediatric panoramic radiographs. The algorithm was trained and tested on a total of 421 panoramic images (7). System performance was assessed using a confusion matrix (7). The AI system was successful in detecting and numbering the deciduous teeth of children with high sensitivity, accurate precision, and good F1 score of 0.9804, 0.9571, and 0.9686, respectively (7). The F1 score is a metric used to assess the performance of machine learning models by combining precision and recall. The F1 score ranges from 0 to 1, with 0 being the worst and 1 being the best. The precise threshold for what is considered a good F1 score depends on the task, model use case, and how much error is tolerated. This Deep-learning-based AI models was a promising tool for the automated charting of deciduous tooth and number in panoramic dental radiographs from children (7).

Kaya et al. (8) in their study used YOLO V4 because of extreme speed and accuracy for object detection. The training data set with 4,045 images was used to train the model, and a randomly selected testing group with 500 images was used to evaluate the performance of the model (8). The proposed CNN method yielded high and fast performance for automated tooth detection and numbering on paediatric panoramic radiographs. Automatic tooth detection could help dental practitioners to save time and also use it as a pre-processing tool for detection of dental pathologies (8).

The Faster Region- Convoluted Neural Network method analyses data set of vast number radiographic images, and yielded highly accurate and faster output of automated tooth detection and numbering, and helps dentists in more accurate diagnosis supernumerary and submerged tooth, to plan appropriate management.

### 3.2 AI based early diagnosis of dental caries in children

Dental caries is the most common dental disease among children and the single most common chronic childhood disease, with considerable economic and quality-of-life burdens (9). It is caused by the dissolution of teeth by acid production due to the metabolism of carbohydrates by certain bacteria (10). Its prevalence is thought to have increased recently in children aged 2–5 years globally, making this age group a global priority action area. If left untreated, dental caries can lead to pain, discomfort, failure to thrive, reduced quality of life, and tooth loss (10). Parents of children with severe dental caries have been shown to take more time off work, report that the child needed more attention, felt guilty, felt stressed, have normal activities disrupted, and have sleep disrupted (11). It is utmost important for early detection of dental caries in children with high risk and support them with preventive measures for optimum oral health.

Sadegh-Zadeh et al. (2) in their study focused on Identifying and targeting high-caries-risk children via dental examinations along with computing methods at an early stage in life, to prescribe strict prevention measures in high-risk groups. Machine learning methods created computer algorithms with multiple correlated parameters was highly successful in the identification and prediction for childhood caries. The best performing machine learning models were Multilayer Perceptron (MLP), Random Forest (RF), and Support Vector Machine (SVM) with RBF kernel, which most accurately classified the presence of risk with an accuracy above 97%.

Another important use of CNN algorithm in Paediatric dentistry is diagnosis of deep caries and pulpitis in periapical radiographs. Zheng et al (11). in their study on AI based automated diagnosis of deep caries and pulpitis, compared 3 different CNN's, of the three ResNet18 along with the clinical data was highly accurate, precise, with high sensitivity and specificity (11). The multi-modal CNN of ResNet18 with the clinical parameters had significant accurate results in the diagnosis of deep caries and pulpitis (11).

Ramos-Gomez et al. (12) and Karhade et al. (13) in their respective studies used applied machine learning for the diagnosis of dental caries using a questionnaire (12, 13). The results demonstrated good performance in the identification and classification of early childhood caries (ECC) (12, 13).

AI based early diagnosis of dental caries in school going children will be an important tool for implementing preventive measures to maintain good oral health.

### 3.3 AI in forensic paediatric dentistry: assessment of bone age

For clinicians, analysing and assessing the age of children during adoptions or illegal stays in some countries, makes knowing the metric age assessment indispensable (13). Due to sexual dimorphism the tooth development is quicker in girls compared to boys. Implementing artificial neural networks to handle medical-related data has gained prominence in recent times, and it also offers better and more efficient diagnostics in various medical conditions (13, 14).

Dental age is generally analysed using one of the two methods, namely the clinical method or the pan tomographic method. The pan tomographic methods of dental age assessment are more precise the mineralization of tooth (14).

Zaborowicz et al. (14) focused on producing a new method for detecting the chronological age of kids and adolescents aged 4–15 years using digital pan tomographic images and neural modelling. This method is simpler, has a near-perfect accuracy, and was one of the first to use pan tomographic images for metric age assessment; however, one of its major limitations is that it does not use 2D photographs and only works with pan tomographic images (13).

Bunyarit et al. (15) developed a new dental maturity ratings based on Demirjian's scores was created through artificial neural network (ANN) computational technique (15). It was observed that the new dental maturity scores may determine the age of Malaysian Chinese children and adolescents (15).

Lee et al. (16) in their study, 18 radio morphometric parameters extracted from panoramic radiographs (PRs) were analysed and focused primarily on developing ML algorithms. It was observed that ML algorithms are more efficient at estimating age compared to traditional estimation (16).

### 3.4 AI based toolkit to assess oral health in children

In developing and underdeveloped countries the priority areas of health are different and oral health is usually ignored. The World Health Organization (WHO) developed an oral health questionnaire (17) for all the age groups to understand about the general oral health concerns (13). Wang et al. (17) developed a tool kit for parents to evaluate the oral health condition of their Children. In addition this questionnaire has physical, mental, and social components that may be associated with oral health.

A study by Gajic et al. (18) analysed the impact of oral health on adolescent quality of life using statistical methods and artificial intelligence algorithms and found that human intuition and machine algorithms both agreed on how the responses should be split. The respondents may be divided into distinctive groups using artificial intelligence algorithms, allowing the finding of information not available with the intuitive classification of respondents by gender.

The machine learning-based toolkit results could be used by all dentists, parents, and even kids to understand an individual's need for oral treatment and to get an idea where someone stands in terms of oral health. It will be necessary for dental education to complement the introduction of clinical AI solutions by encouraging digital literacy among those who will work in the dental field in the future (13).

### 3.5 AI in early detection of dental plaques in children

Dental plaque is a condition where a membrane of bacterial commensals are formed on the surface of the teeth, especially along the gingival margins leading to gingivitis. It may be challenging even for experienced dentists to detect dental plaques in their early stages (19). Other disadvantages, more on the aesthetic side, include their unpalatable taste and retained stains on the lips and oral membrane (19). You et al. (19) in their study successfully presents a novel AI model for detecting dental plaque on primary teeth by the caretakers. An AI based algorithm was developed which achieved clinically acceptable accurate prediction levels for detecting dental plaque on primary teeth in comparison with an experienced paediatric dentist (19).

Joesph et al. (20) developed a Laser Induced Auto Fluorescence (LIAF) based probe connected to AI program which could detect different grades of plaque on tooth surfaces including the clinically invisible type belonging to grade-1 from the 404 nm laser-induced LIAF spectral intensity ratio F510∕F630. The Fluorescence Ratio Reference Standard (FRRS) developed by Joseph et al. (20) has the potential benefit of quantifying even minute amounts of plaque in real time, thereby achieving easier plaque detection for the clinician and better plaque control for the patient.

AI based algorithms may be developed and used in early diagnosis and aids prediction of accurate diagnosis in children (21). The use of the newer technology of CNN and other deep learning methods along with appropriate algorithms is largely dependent on the quality and quantity of data sets that are used in each application. The limitations of this article is that it is a very concise review about a new emerging technology. A detailed discussion about the various usage of different machine learning and deep learning techniques will be useful for better understanding and initiate further research studies.

## 4 Conclusion

The AI based tools can be widely used in the various fields of automated diagnosis and prediction in paediatric dentistry. The limitations in using these tools can be overcome by using it as a complementary aid to dentists in their clinics. AI technology may be used in the automated diagnosis in paediatric dentistry in the various conditions as explained above and it requires further research to be translated into everyday practice. The field of AI, deep machine learning and CNN's is an upcoming and newer area, with new developments this will open up areas for more sophisticated algorithms in multiple layers to predict accurately comparable to experienced Paediatric dentists.
